# Assessment of Bowel Urgency in Ulcerative Colitis: Current Measures and Future Directions

**DOI:** 10.1016/j.gastha.2026.101086

**Published:** 2026-08-11

**Authors:** Marla Dubinsky, Richard Moses, Laurent Peyrin-Biroulet, Simon Travis, David T. Rubin, Alissa Walsh, Alison Potts Bleakman, Charles Owen, Michael Camilleri

**Affiliations:** 1 Susan and Leonard Inflammatory Bowel Disease Clinical Center, Icahn School of Medicine at Mount Sinai, New York, New York; 2 Eli Lilly and Company, Indianapolis, Indiana; 3 Department of Gastroenterology, University Hospital of Nancy, University of Lorraine, Nancy, France; 4 Translational Gastroenterology Unit, Experimental Medicine Division, Nuffield Department of Medicine, John Radcliffe Hospital, University of Oxford, Oxford, UK; 5 Inflammatory Bowel Disease Center, University of Chicago Medicine Inflammatory Bowel Disease Center, Chicago, Illinois; 6 Translational Gastroenterology Unit, NIHR Oxford Biomedical Research Centre, Nuffield Department of Medicine Experimental Medicine Division, University of Oxford, Oxford, UK; 7 Clinical Enteric Neuroscience Translational and Epidemiological Research, Mayo Clinic, Rochester, Minnesota

**Keywords:** Adults, Fecal Incontinence, Inflammatory Bowel Diseases, Patient-Reported Outcomes, Ulcerative Colitis

## Abstract

Bowel urgency (BU), a burdensome ulcerative colitis (UC) symptom, impacts quality of life. It is associated with higher levels of disease severity, corticosteroid use, hospitalization, and colectomy. Despite its prevalence and clinical significance, BU is underassessed in clinical practice and infrequently included as a clinical trial end point. This review evaluates the strengths and limitations of available patient-reported outcome (PRO) measures assessing BU in UC to inform future directions for its assessment. A literature review was conducted using PubMed, Google Scholar, and Embase to identify publications through October 31, 2024, using the key terms “ulcerative colitis assessment” and “bowel urgency.” Publications assessing BU in UC using PRO scales developed according to Food and Drug Administration guidance were included. Five tools met the inclusion criteria. The Urgency Numeric Rating Scale provides a simple and time-efficient tool for clinical use, while multi-item tools such as the Symptoms and Impacts Questionnaire for Ulcerative Colitis and the Ulcerative Colitis-Symptom Questionnaire include comprehensive symptom tracking but impose greater burdens for clinical practice. Diary-based tools, including the Ulcerative Colitis-Patient-Reported Outcomes/Signs and Symptoms Diary and Patient-Reported Outcomes-Ulcerative Colitis Diary, allow detailed symptom monitoring in clinical trials but are less practical for routine clinical use. Some dimensions of BU are missing from the tools reviewed. Incorporating validated BU measurement into routine clinical care and research is essential for advancing patient-centered, comprehensive disease management and improving outcomes in patients with UC.

## Graphical Abstract

**Figure:**
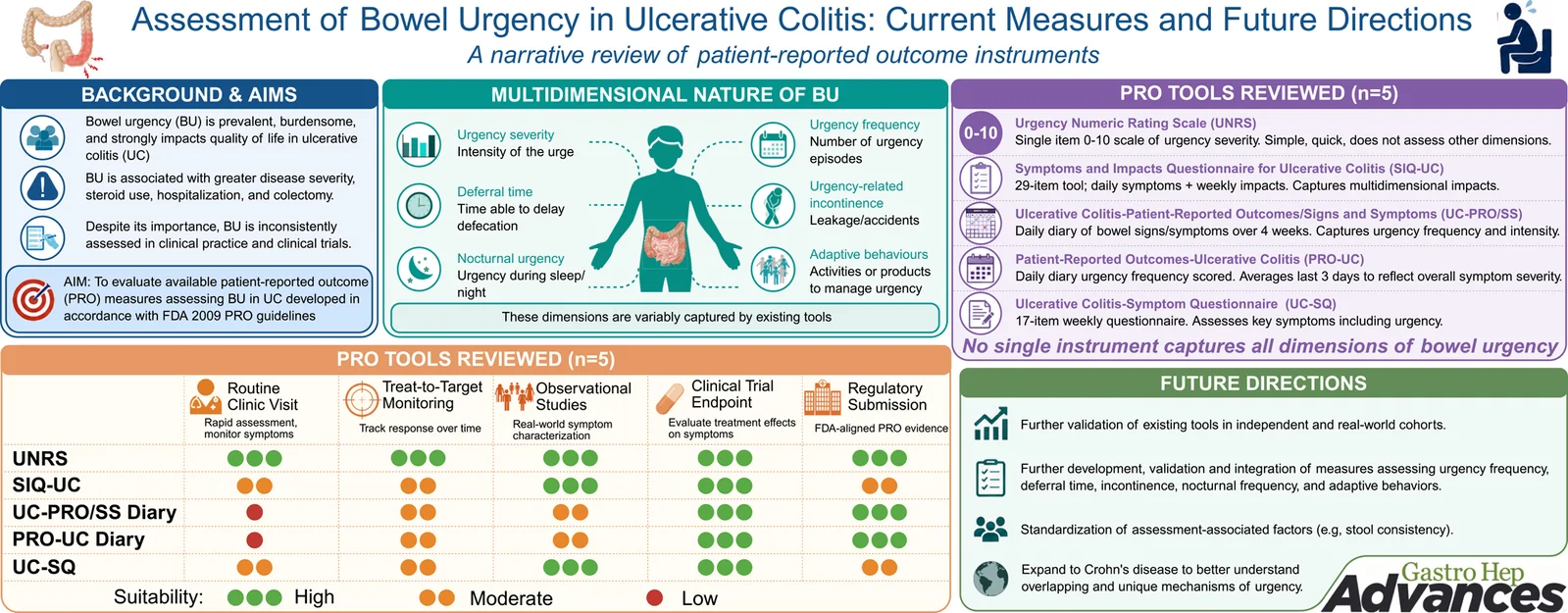

## Introduction

Ulcerative colitis (UC) is a chronic, idiopathic inflammatory bowel disease (IBD) characterized by mucosal inflammation of the large intestine, extending proximally from the rectum.^1^^,^^2^ Patients with UC can experience daily disruptive symptoms of varying severity, with bowel urgency (BU) identified as one of the most frequent and bothersome symptoms, exerting the greatest impact on quality of life.^3^, ^4^, ^5^, ^6^, ^7^ Despite its significant burden, BU is often underrecognized in clinical practice, as patients may feel embarrassed to discuss it, and health-care providers may not proactively address it.^8^, ^9^, ^10^

BU is generally defined as the sudden or immediate need to have a bowel movement.^11^ BU encompasses multiple dimensions that should be distinguished during assessment, including urgency severity (the perceived intensity of the urge), urgency frequency (the number of urgency episodes over a specified period), and stool deferral time (the duration for which defecation can be postponed after the urge arises).^11^ These dimensions may contribute independently to symptom burden and quality-of-life impairment.

Clinical research has demonstrated that BU is a distinct symptom in UC, independent of stool frequency and rectal bleeding.^1^ Emerging evidence suggests that, besides rectal bleeding, symptoms such as abdominal pain and BU may better reflect clinical disease activity than stool frequency.^12^ Recently registered clinical trials in adults with moderate-to-severe UC have been encouraged by regulatory agencies to incorporate patient-reported outcome (PRO) measures that assess the impact of investigational drugs on symptoms such as BU, beyond established end points such as stool frequency and rectal bleeding.^13^^,^^14^

Despite recognition that BU should be measured and controlled in UC, there is no global consensus on the best methods to assess or measure it in clinical trials or routine practice. A systematic, standardized, validated approach to defining and assessing BU in patients with UC would facilitate BU inclusion as an outcome in UC clinical trials and routine practice.^15^

This review aims to evaluate the strengths and limitations of current BU assessment scales in UC. It summarizes their use, presents evidence supporting their validation, and offers perspectives on future directions for improving and standardizing BU assessment in clinical trials and practice.

## Methods

This review evaluated PRO scales for assessing BU in UC, following the Preferred Reporting Items for Systematic Reviews and Meta-Analyses guidelines. The search targeted peer-reviewed literature, primary studies, abstracts, systematic reviews, and meta-analyses examining PRO scales for UC, specifically BU.

Eligible publications involved patients with UC in whom BU was assessed using PRO scales developed or validated in accordance with the US Food and Drug Administration (FDA) 2009 PRO guidance. Dissertations, commentaries, editorials, and unpublished reports or manuscripts were excluded. Literature searches were conducted in PubMed, Google Scholar, and Embase through October 31, 2024. Search terms included “ulcerative colitis,” “ulcerative colitis assessment,” “bowel urgency,” “fecal urgency,” “urgency,” “tool,” “instrument,” “scale,” “FDA PRO guidance,” and “patient-reported outcomes.” The search was limited to English-language articles involving adult humans. References for identified articles were manually reviewed to identify additional relevant citations.

Titles and abstracts identified through the literature search were screened for relevance to the assessment of BU in UC. Full-text review was subsequently performed for potentially eligible publications. Publications were evaluated according to predefined eligibility criteria, including assessment of BU in adults with UC using PRO instruments developed or validated in accordance with FDA PRO guidance. Instruments were excluded if they were not specific to UC, did not include assessment of BU, were not PRO measures, were not developed according to FDA guidance, or lacked sufficient published information regarding development or validation. Because the objective of this review was to summarize currently available BU assessment instruments rather than perform a quantitative evidence synthesis, formal duplicate screening and risk-of-bias assessment were not conducted. Figure shows the study selection flowchart.

**Figure:**
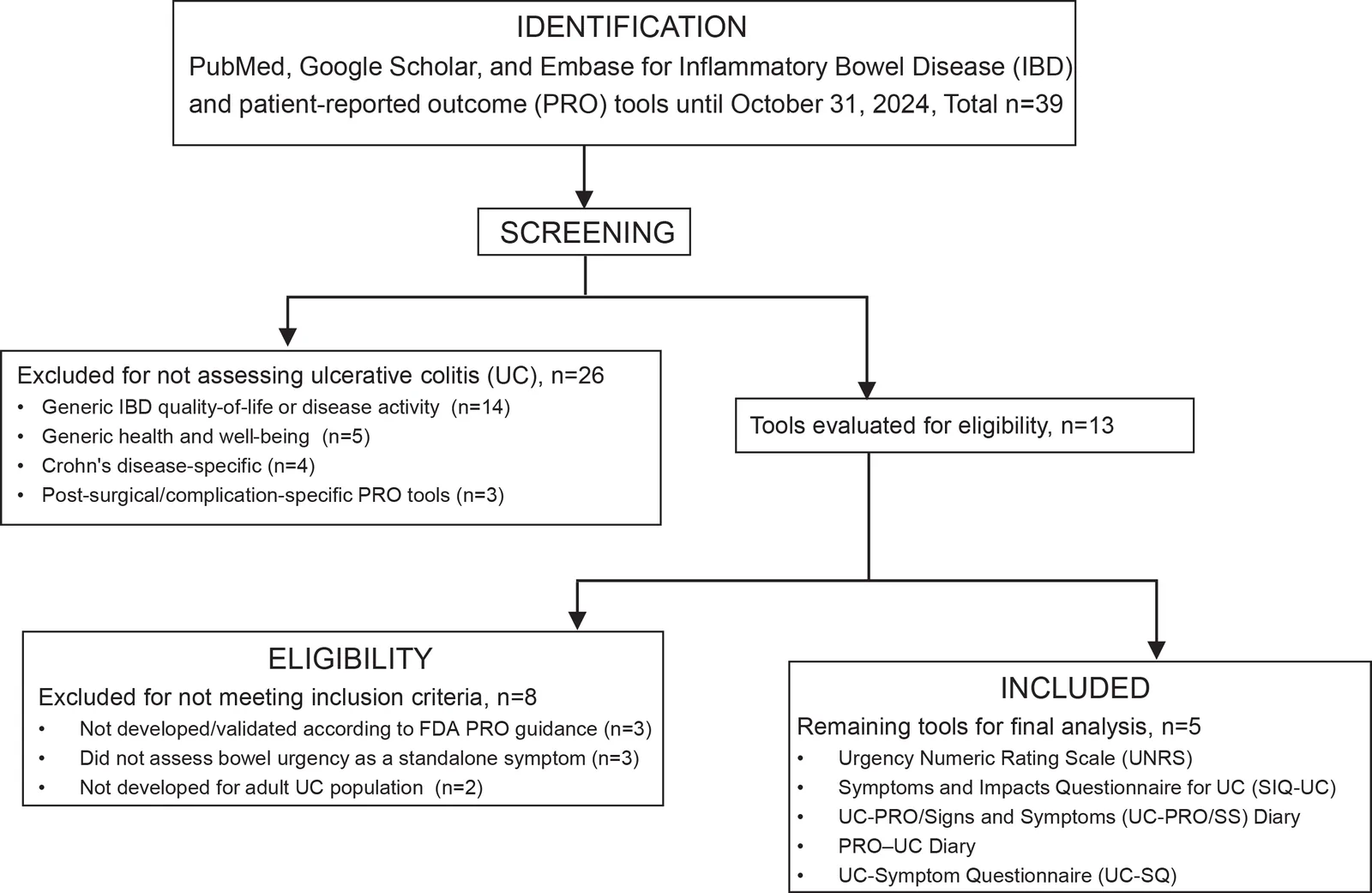
Process map for literature review.

### Current Guidelines and Recommendations

Recent guidelines emphasize addressing BU in clinical practice and research. The American College of Gastroenterology clinical guidelines for adult UC identify BU as a key symptom for assessing disease severity, alongside abdominal pain, cramping, and weight loss.^2^ The guidelines recommend using instruments such as the proposed American College of Gastroenterology UC Activity Index and the Simple Clinical Colitis Activity Index (SCCAI), both of which include BU as a component of measuring disease activity.^2^ The widely used patient-reported SCCAI questionnaire, adapted from the SCCAI, assesses BU through questions such as “During the previous week, were you able to hold up your stool for 15 min or longer, when you felt the urge to use the toilet?” and “During the previous week, did you have to make adjustments to your activities, to ensure that there was a toilet nearby?”^15^^,^^16^ However, these indices were not developed according to FDA guidance.^2^^,^^16^, ^17^, ^18^, ^19^, ^20^ The guidelines further recommend that treatment goals should prioritize control of BU, alongside normalization of stool frequency and the absence of rectal bleeding.^2^

Historically, BU has not been addressed by outcome measures commonly used in clinical trials and is inconsistently assessed in UC studies. In its April 2022 draft guidance, the FDA recommends the modified Mayo Score (mMS) as the primary end point for evaluating clinical remission in UC trials; however, the mMS focuses on stool frequency, rectal bleeding, and endoscopic assessment.^13^ Recognizing the limitations of mMS in capturing other relevant patient experiences, the FDA advocates for the inclusion of additional end points to assess investigational drug effects on patient-reported symptoms such as abdominal pain and BU.^13^ The guidance further recommends using fit-for-purpose PRO scales to evaluate these additional symptoms.^13^ Similarly, recent international consensus statements support including BU, alongside stool frequency and rectal bleeding, as a core outcome measure for UC disease activity in trials.^21^^,^^22^

### Multidimensional Aspects of Bowel Urgency in Ulcerative Colitis

BU in UC is a multidimensional symptom, with severity influenced by deferral time, frequency of BU events, stool consistency, concomitant symptoms, and adaptive behaviors.^23^ Most commonly, BU in clinical trials has been assessed using a binary measure, categorizing urgency as present or absent. While this measure is straightforward, patients with UC prefer BU scales that distinguish varying levels of severity and urgency rather than a yes-or-no response.^24^ The binary approach fails to capture the severity or multidimensional nature of BU^25^ and, consequently, may overlook meaningful improvements that fall short of complete resolution (ie, absence of urgency).^25^ A crosswalk analysis by Dubinsky et al further illustrates these limitations: mirikizumab response rates for “absence of urgency,” measured by a binary questionnaire, corresponded approximately to scores <3 on the 11-point Urgency Numeric Rating Scale (UNRS), not exclusively to the score 0. This reinforces the need for more granular tools for assessment of BU in research and practice.^25^

Deferral time, the length of time a patient can delay defecation after the urge arises, can be very short. Up to 54% of patients with UC report a deferral time of ≤5 min, with some patients reporting <30 s in severe cases.

BU frequency, defined as the number of BU events over a given time period, further reveals UC burden. In the Communicating Needs and Features of IBD Experiences study, 78.9% of the US patients (n = 123) and 70.8% of the European patients (n = 250) with a history of BU experienced urgency weekly over the past 3 months. Similarly, among those receiving advanced therapy (US, n = 91; Europe, n = 126), 80.2% and 73.9%, respectively, reported weekly urgency.^10^ BU is often accompanied by symptoms such as abdominal pain, fatigue, and abdominal cramping.^26^ Patients frequently adopt a range of adaptive behaviors to manage or prevent incontinence associated with BU,^11^^,^^27^ including protective measures such as adult diapers and pads.^10^^,^^11^ These behaviors highlight the need to address not only BU’s physical manifestations but also its social impacts.

Stool consistency is also related to BU and remains underrepresented in current BU assessment instruments. In a cross-sectional study of patients with UC, softer or unformed stools were associated with moderate-to-severe urgency, alongside increased bowel movement frequency, rectal bleeding, and abdominal pain.^28^ A review of fecal urgency in IBD further reported that diarrhea is often linked to urgency and may worsen it through mechanisms such as rapid stool transit and reduced stool consistency, both of which increase rectal stimulation.^15^

These findings suggest that stool consistency may contribute to the experience of BU and warrant further investigation. Future studies could evaluate whether the incorporation of standardized stool form assessments, such as the Bristol Stool Form Scale,^29^ enhances the characterization of BU evaluation in UC. Including stool form may enhance the accuracy of symptom characterization and improve the sensitivity of PRO measures in clinical trials and routine care.

### Bowel Urgency Assessment Measures in Patient-Reported Outcome

The recommendation to incorporate PRO in the assessment of BU in UC necessitates validated, sensitive, and patient-centered tools for measurement in clinical trials and routine practice.^30^^,^^31^ Several PRO tools have been developed in accordance with FDA 2009 PRO guidelines,^32^ including the UNRS, Symptoms and Impacts Questionnaire for Ulcerative Colitis (SIQ-UC), Ulcerative Colitis-Patient-Reported Outcomes/Signs and Symptoms (UC-PRO/SS) Diary, Patient-Reported Outcomes-Ulcerative Colitis (PRO-UC) Diary, and the Ulcerative Colitis-Symptom Questionnaire (UC-SQ) (Table 1).^33^, ^34^, ^35^, ^36^, ^37^ While the 2009 guidance established the general principles for the development of PRO, the FDA 2022 guidance extends these principles with disease-specific recommendations for UC. Additionally, the 2022 FDA guidance document advises that PRO scales for UC include patient-derived qualitative data to ensure the relevance of selected symptoms, clarity for patient use, and evidence of baseline symptom severity and clinically meaningful improvements.^13^

**Table 1:** Comparison of Patient-Reported Outcome Measures That Include Bowel Urgency for Ulcerative Colitis Developed in Accordance With Food and Drug Administration Guidance

	Standalone BU measure	BU in composite measures			
	UNRS^33^	SIQ-UC^34^	UC-PRO/SS diary^35^	PRO-UC diary^36^	UC-SQ^37^
Content	Single-item symptom 0–10 urgency scale	4 symptoms and 6 impact domains	9 symptoms	6 symptoms	17 symptoms
Number of survey items	1	29	9	6	15 (+2 additional non-scored)
Geographical region	United States	United States and Canada	United States	United States	United States
Validation method	Qualitative interviews, 2-wk diary;^38^ multicenter phase 3 trial, psychometric analysis^33^	Concept elicitation, cognitive interviews	Focus groups, 4-wk observational study	Concept elicitation, cognitive interviews	Phase 2b trial data, psychometric testing
Psychometric validity	Yes	No	Yes	No	Yes
Redundancy with mMS	No	Yes	Yes	Yes	Yes
Encompasses multidimensional aspect of BU	Partially	Partially	Partially	Partially	No
Key strengths	Single-item scale focused on urgency; quick, low burden, no repetition with other measures; clinically meaningful threshold established for improvement and remission; validation for extended recall periods	Comprehensive; covers impact domains	Reliable for daily symptom tracking and comprehensive severity assessment	Comprehensive; assesses multiple UC symptoms	Psychometric validation, trial tested for severity; clinically meaningful threshold established for improvement
Key limitations	Simplifies the multidimensional aspect of BU; not comprehensive for overall UC disease severity	Higher burden for completion, potential for overlap with other PROs; complex and lengthy; psychometric validation in progress, lacks full trial-based validation	Higher burden for completion, potential for overlap with other PROs; complex daily diary format; limited to moderate-to-severe UC	Higher burden for completion, potential for overlap with other PROs; early validation stage, needs further psychometric analysis	Higher burden for completion, potential for overlap with other PROs, validated in moderate-to-severe UC; does not apply to mild cases

BU, bowel urgency; mMS, modified Mayo Score; PRO, patient-reported outcome; PRO-UC, Patient-Reported Outcome-Ulcerative Colitis; SIQ-UC, Symptoms and Impacts Questionnaire for Ulcerative Colitis; UC, ulcerative colitis; UC-PRO/SS, Ulcerative Colitis-Patient-Reported Outcomes/Signs and Symptoms; UC-SQ, Ulcerative Colitis-Symptom Questionnaire; UNRS, Urgency Numeric Rating Scale.

### Urgency Numeric Rating Scale: A Standalone Measure of Bowel Urgency

The UNRS is a single-item PRO measure designed to evaluate the severity of BU in adults with UC. The scale ranges from 0 to 10, with 0 representing “no urgency” and 10 indicating “worst possible urgency.” Patients are prompted to respond to the question, “How severe was your urgency (sudden or immediate need) to have a bowel movement?” Three recall period options have been validated: in the past 24 h, 3 days, and 7 days.^33^^,^^38^^,^^39^ For the 24-hour recall period, the severity of urgency is reported over 24 h, and a weekly average score can be calculated as the mean (to the nearest whole number) of daily scores over 7 days.^33^^,^^38^ The 3-day and 7-day recall versions do not require any calculations.^39^^,^^40^ Higher scores indicate greater BU severity. The simplicity of the UNRS enhances patient usability by avoiding specific descriptors that may not apply to all individuals, thereby capturing the broad spectrum of urgency experiences.

The UNRS has several strengths as a tool for assessing BU in UC. Its brief, validated format is well-suited for clinical trials and routine practice, where regular assessment can facilitate monitoring and treatment response over time.^33^ The absence of specific units allows patients to report overall urgency severity based on their individual experiences, a format shown to be effective for subjective symptoms such as abdominal pain in irritable bowel syndrome.^41^ The 24-hour recall daily diary option also captures the relapsing-remitting nature of UC.^33^ The UNRS is a simple, single-item tool that can potentially support high feasibility and patient compliance.

However, the simplicity of the UNRS also represents an important limitation. As a single-item measure focused on urgency severity, it does not directly assess other clinically relevant dimensions of BU, including urgency frequency, stool deferral time, urgency-related incontinence, nocturnal urgency, adaptive behaviors used to prevent accidents, or the impact of urgency on daily functioning and quality of life. Also, full psychometric validation has only been established for the weekly mean UNRS scores derived from daily assessments with a 24-hour recall, as intended for clinical trials. Consequently, although the UNRS provides an efficient measure of BU, it may not fully characterize the multidimensional burden of BU experienced by patients with UC, and formal psychometric testing for these longer recall periods is still under development.^38^, ^39^, ^40^

### Bowel Urgency in Composite Measures

#### Symptoms and impacts questionnaire for ulcerative colitis

The SIQ-UC is a 29-item questionnaire developed to evaluate UC symptoms and their impacts. It includes 3 modules, a daily bowel movement report, a daily symptom diary, and a weekly impact assessment, completed over 7 days.^34^ This structure captures daily fluctuations and weekly trends in symptom severity, providing a detailed profile of the physical and psychosocial burden of UC. The SIQ-UC evaluates 4 symptom domains: gastrointestinal, pain/discomfort, nutrition-related, and energy-related. The questionnaire specifically assesses daily BU by asking patients, “How strong was your urge to use the restroom before this bowel movement?” with response options ranging from “not strong at all” to “extremely strong.” Additionally, the following 6 impact domains are assessed: emotional, daily performance, lifestyle/activities, social functioning, dietary, and additional/overall quality of life. Together, these domains capture both the clinical and quality-of-life effects of UC.

The SIQ-UC has notable strengths, particularly in its impact section, which evaluates the effects of UC on patients’ functional abilities and emotional well-being relative to their disease status. This multidomain design provides a comprehensive evaluation, capturing both symptom severity and improvements in functionality and psychological health.

The SIQ-UC scale has some limitations. Ongoing psychometric validation is required to confirm reliability and responsiveness. Furthermore, recruitment during the scale development was restricted to North America, which may limit the generalizability of the findings to broader IBD populations. These limitations should be considered when interpreting results, and further validation studies are recommended to enhance the scale’s robustness.

#### Ulcerative colitis-patient-reported outcomes/signs and symptoms diary

The UC-PRO/SS Diary is a PRO measure designed to assess gastrointestinal signs and symptoms in patients with moderate-to-severe UC for use in clinical trials.^35^ It comprises 2 scales, Bowel Signs and Symptoms (6 items) and Abdominal Symptoms (3 items), both psychometrically validated and able to differentiate between varying levels of disease severity.

BU is evaluated in the UC-PRO/SS through items measuring both the frequency and intensity of the immediate need to defecate. Response options capture presence (yes/no) as well as severity or frequency, with scores ranging from 0 (none or not at all) to 4 (always or very severe). The comprehensive range of symptoms assessed, patient-centered design, and ability to differentiate symptom severity establish the UC-PRO/SS as a valuable tool for both clinical practice and research in UC.^35^

The UC-PRO/SS diary limitations include the fact that its 4-week observation period may not fully capture the variability or fluctuations of UC symptoms. Baseline endoscopy was not required, leaving the inflammatory status of the colon or rectal mucosa at time of participation unknown. Additionally, the study sample did not include patients experiencing an acute flare, limiting the scale’s evaluation in capturing symptom severity during peak disease activity. Several items, such as leakage and the presence of blood or mucus in stools, consistently received the lowest scores, suggesting the validation was performed primarily in patients with mild disease burden, and its performance in more severe cases remains unclear.^35^

#### Patient-reported outcomes-ulcerative colitis diary

The PRO-UC Diary is a PRO measure for adults with moderate-to-severe UC. The diary evaluates specific symptoms within a 24-hour recall period, including stool frequency, rectal bleeding (severity and frequency), diarrhea, frequency of rectal urgency, and abdominal pain.^36^

BU is quantified by recording the number of bowel movements accompanied by urgency, which are then converted to a 0 to 10 scale: 0 to 2 events score 0, 3 to 5 events score 2.5, 6 to 8 events score 5.0, 9 to 11 events score 7.5, and 12 or more events score 10. Higher scores indicate greater severity of signs and symptoms. The Total Signs and Symptoms score is calculated as the average of the symptom scores over the last 3 days of available data (except for rectal bleeding), providing a standardized measure of overall symptom severity. This frequency-based approach allows the PRO-UC Diary to capture both the intensity and daily variability of UC symptoms, facilitating a comprehensive assessment of urgency and other core symptoms in clinical research.

The PRO-UC Diary has several key strengths, particularly its daily reporting structure, which enables detailed tracking of symptom fluctuations, providing a robust measure of short-term disease activity. Additionally, averaging symptom frequency over a 3-day period helps mitigate recall bias, supporting the accuracy of symptom severity reporting. Developed through patient interviews, the final 6-item diary underwent content validation, confirming its effectiveness in capturing core UC symptoms.^36^

However, certain limitations should be noted. The final electronic version of the diary was only tested by 4 participants, potentially limiting insights into its usability and functionality in larger clinical settings. Furthermore, the absence of data on disease location in these initial evaluations may restrict understanding of how symptom presentation and severity may vary across different UC subtypes. Although the diary demonstrated strong initial validity, further psychometric testing is recommended to confirm its reliability, responsiveness, and ability to capture the impact on quality of life.^36^

#### Ulcerative colitis-symptom questionnaire

The UC-SQ is a 17-item questionnaire designed to assess key UC symptoms. Of these, 15 are scored items targeting UC-specific symptoms such as bowel movement frequency, abdominal pain, rectal pain, urgency, fatigue, and diarrhea.^37^ BU is assessed with the following item: “During the past week, did you experience a sudden or intense need to have a bowel movement?”^37^ Two additional items, joint pain and constipation, are included as non-scored informational items. Each question uses a 5-point Likert-type scale to measure either symptom frequency (0 = never to 4 = always) or intensity (0 = not at all to 4 = very much), reflecting symptom severity over the past week.^37^

The total UC-SQ score, derived from the 15 scored items, ranges from 0 to 68, with higher scores indicating greater symptom severity. UC-SQ is a psychometrically validated scale developed with input from both patients and clinicians. It is responsive to changes in symptoms over time and includes a threshold for clinically meaningful improvement, enhancing its interpretability in clinical evaluations.^37^

Several limitations in the development and validation of the UC-SQ should be noted. The study sample lacked baseline diversity in disease severity in the development stage, with most participants in remission or exhibiting mild disease, thus limiting generalizability. The UC-SQ was also developed in an English-speaking US population, necessitating further validation for cultural and linguistic adaptability internationally. Although educational levels of participants varied, literacy was not formally assessed. Additionally, the absence of a baseline patient global impression of severity also limited direct tracking of symptom changes over time. Finally, the test-retest reliability assessment, conducted 2 weeks posttreatment, may have influenced responses; ideally, this assessment should be performed closer to baseline.^37^

### Minimal Clinically Important Difference in Ulcerative Colitis-Specific Patient-Reported Outcome Measures

As discussed above, several UC-specific PRO instruments have been developed to capture symptom severity and disease impact from the patient’s perspective, particularly among adults with moderate-to-severe UC. Notably, the UNRS, UC-PRO/SS Diary, and UC-SQ have minimal clinically important difference (MCID) thresholds. For the UNRS, at least a 3-point reduction from baseline represents meaningful clinical improvement, while a posttreatment score of 0 or 1 reflects the BU remission threshold closely associated with clinical, endoscopic, and histologic remission.^38^ The UC-PRO/SS Diary, which encompasses bowel and functional domains, has MCID thresholds of 6 and 2 points, respectively.^42^ For the UC-SQ, a 10-point reduction has been established as the threshold for clinically meaningful improvement, signifying a potential reduction in symptoms and associated improvements in patient quality of life.^37^ In contrast, MCID values have not yet been established for the SIQ-UC and PRO-UC Diary. While multiple UC-specific PRO instruments show promise for capturing meaningful change, only a subset currently have thresholds to guide interpretation in clinical trials and routine practice.

### Recommendations and Future Outlook for the Assessment of Bowel Urgency in Ulcerative Colitis

Approximately 25%–50% of surveyed patients with UC report experiencing BU at least once daily.^3^^,^^43^ The persistent and disruptive nature of BU has been linked to reduced daily functioning, diminished health-related quality of life, and an increased risk of depression, anxiety, and fatigue,^3^^,^^4^^,^^8^^,^^28^^,^^43^^,^^44^ often causing more distress than stool frequency and rectal bleeding.^45^, ^46^, ^47^, ^48^ BU has also been associated with a higher risk of colectomy, corticosteroid use, and hospitalization.^44^ Clinically meaningful improvement in BU has been recognized by regulatory agencies as a benefit of advanced therapy, such as monoclonal antibodies, and included in product labels.^49^^,^^50^ Additionally, the Community, Outreach, Research and Education in IBD initiative identified BU as a core outcome because of its substantial impact on quality of life.^14^ Separately, an expert panel commissioned to appraise disease control in UC using the Delphi process unanimously agreed (9 of 9 members) that patient-reported BU should be part of the comprehensive disease control assessment in UC.^51^

The reviewed scales for assessing BU in UC offer complementary strengths across clinical practice and research settings, varying in scope, complexity, and intended use. The 3-day and 7-day recall period versions of the UNRS provide simple and feasible assessments of urgency severity and may be particularly useful in routine clinical practice, treat-to-target monitoring, and studies where minimizing patient burden is important. The UNRS also aligns with FDA guidance encouraging the development of additional PRO scales to capture patient-relevant concepts beyond those covered by the mMS, making it a practical complementary tool for clinical trials and observational studies. Since discrepancies between patient-reported disease activity and endoscopic findings are common in UC, and endoscopic assessment has never been validated as a predictor of BU,^12^ the UNRS offers meaningful clinical insight without the need for endoscopic evaluation. However, because it focuses primarily on urgency severity, the UNRS does not fully capture other dimensions of BU. In settings where a more comprehensive assessment is desired, multidimensional instruments may provide additional clinically relevant information.

In contrast, the composite assessment tools discussed are not explicitly focused on BU, as they incorporate the assessment of other symptoms. Consequently, these tools may be less targeted, take longer to complete, and may not efficiently capture the distinct severity or impact of BU. Although these multi-item scales address BU, they contain multiple items overlapping with traditional measures such as the mMS, which prioritizes stool frequency and rectal bleeding as primary end points in UC trials. This redundancy may increase the assessment burden without providing substantial new insights into disease activity.

The SIQ-UC provides a more comprehensive assessment of UC symptoms and their impact on daily life, making it valuable for in-depth patient evaluations, although its length may limit feasibility for routine use. The UC-PRO/SS Diary and the PRO-UC Diary, designed to capture daily symptom fluctuations, are beneficial in clinical trials where detailed daily monitoring is essential but may be burdensome in routine clinical practice. The UC-SQ is well-suited for a comprehensive assessment in clinical trials, with practicality for occasional use in clinical settings, though it does not specifically focus on BU.

The choice of assessment tools should align with specific clinical or research objectives. No currently available instrument comprehensively captures all clinically relevant dimensions of BU. The UNRS may be useful when rapid assessment of urgency severity is desired, whereas multidimensional instruments such as the SIQ-UC, UC-SQ, UC-PRO/SS Diary, and PRO-UC Diary may provide broader information regarding symptom burden, functional impact, and associated symptoms (Table 2). Emerging measures evaluating urgency frequency, stool deferral time, and behavioral responses may further enhance the assessment of BU in future clinical practice and research.

**Table 2:** Suggested Applications of Available Bowel Urgency Assessment Instruments in Ulcerative Colitis

Instrument	Routine clinic visit	Treat-to-target monitoring	Observational studies	Clinical trials	Regulatory end points
UNRS^33^^,^^38^, ^39^, ^40^	High	High	High	High	High
SIQ-UC^34^	Moderate	Moderate	High	High	Moderate
UC-PRO/SS diary^35^	Low	Moderate	Moderate	High	High
PRO-UC diary^36^	Low	Moderate	Moderate	High	High
UC-SQ^37^	Moderate	Moderate	High	High	Moderate

PRO-UC, Patient-Reported Outcomes-Ulcerative Colitis; SIQ-UC, Symptoms and Impacts Questionnaire for Ulcerative Colitis; UC-PRO/SS, Ulcerative Colitis-Patient-Reported Outcomes/Signs and Symptoms; UC-SQ, Ulcerative Colitis-Symptom Questionnaire; UNRS, Urgency Numeric Rating Scale.

Note: Selection should be based on the intended purpose of assessment, patient burden, and desired level of symptom characterization. No currently available instrument comprehensively captures all dimensions of bowel urgency.

From a practical perspective, the selection of a BU assessment instrument should be aligned with the intended clinical or research objective. In routine clinical practice, brief instruments with minimal patient burden, such as the UNRS, may facilitate rapid symptom assessment and longitudinal monitoring. For treat-to-target approaches, repeated measurement of urgency severity using a simple tool may support assessment of treatment response over time. In observational studies and clinical trials, more comprehensive instruments such as the SIQ-UC, UC-PRO/SS Diary, PRO-UC Diary, and UC-SQ may provide additional information regarding symptom burden, associated symptoms, and the impact of disease on daily functioning. Regulatory-focused studies may benefit from instruments developed and validated according to FDA PRO guidance, particularly when evaluating treatment effects on patient-relevant symptoms. Ultimately, the optimal assessment strategy should balance feasibility with the need to capture clinically meaningful dimensions of BU.

Assessments of BU should first prioritize measuring and tracking symptom severity, with the potential to expand toward capturing the broader impact of BU on patients’ lives. For patients affected by BU, key areas for consideration include sleep disturbances and nocturnal urgency, measurement of stool deferral time, tracking of BU frequency, urge-related fecal incontinence, and the use of absorbent products to try to mitigate its impact. Two new BU assessment tools, BU Frequency and Stool Deferral Time, are currently being explored for use alongside the validated UNRS and included clinical end points.^52^ In addition, the recently developed URGENT index, an IBD-specific BU index incorporating emotional and behavioral responses to BU, has shown correlation (summarized by the Spearman correlation coefficient of 0.77) with the UNRS. Prospective validation is planned to assess its clinical utility and reliability.^53^ Together, these measures will provide a more comprehensive understanding of BU and its response to treatment. Integrating these factors into BU assessments would better capture symptom burden and support strategies to improve the quality of life for individuals with UC.

An additional consideration when interpreting the available evidence is that several BU assessment instruments and validation studies have been developed within the context of industry-sponsored therapeutic development programs. These collaborations have contributed substantially to the advancement of patient-centered outcome measurement and have supported the incorporation of BU into clinical research and regulatory discussions. However, broader validation in independent cohorts and real-world clinical settings remains important to further establish the generalizability, responsiveness, and applicability of these instruments across diverse patient populations and health-care environments.

Although this review focuses on UC, BU is also an important symptom in Crohn's disease and may similarly affect quality of life and daily functioning.^54^ Future research is needed to better understand the mechanisms and assessment of BU across populations with IBD and to determine whether existing or emerging urgency assessment tools are applicable beyond UC.^39^^,^^55^^,^^56^ Standardized instruments might even be applied (after further validation) to functional diarrhea, irritable bowel syndrome with diarrhea, and bile acid diarrhea, since urgency significantly impacts the quality-of life.^57^

Finally, although some PRO instruments for UC include items related to stool consistency, the methods used are heterogeneous and lack standardization. Future research should evaluate whether standardized stool form measures, such as the Bristol Stool Form Scale, provide incremental value for the assessment of BU and related symptoms.

## Conclusion

BU is a common and burdensome symptom that impairs quality of life for patients with UC, affecting their professional, educational, and social functioning. Although several validated instruments are available, each captures different aspects of BU and no single tool currently provides a comprehensive assessment of all clinically relevant dimensions. Continued refinement and validation of BU assessment tools remain important areas for future research to ensure their broad applicability and facilitate their integration into routine clinical care and research studies. Facilitating open discussions about BU between patients and health-care providers during routine visits can foster collaborative care and improve treatment satisfaction. Moreover, incorporating BU-specific metrics into clinical practice, research, and trials is essential for advancing patient-centered approaches in the management of UC.
